# Assessment of Cleaning and Disinfection Practices on Pig Farms across Ten European Countries

**DOI:** 10.3390/ani14040593

**Published:** 2024-02-11

**Authors:** Iryna Makovska, Ilias Chantziaras, Nele Caekebeke, Pankaj Dhaka, Jeroen Dewulf

**Affiliations:** 1 Veterinary Epidemiology Unit, Faculty of Veterinary Medicine, Ghent University, Salisburylaan 133, 9820 Merelbeke, Belgium; ilias.chantziaras@ugent.be (I.C.); pankaj.dhaka2@gmail.com (P.D.); jeroen.dewulf@ugent.be (J.D.); 2 Biocheck.Gent BV, 8720 Dentergem, Belgium; nele.caekebeke@biocheckgent.com; 3 Centre for One Health, College of Veterinary Science, Guru Angad Dev Veterinary and Animal Sciences University, Ludhiana 141004, India

**Keywords:** biosecurity, cleaning, disinfection, Biocheck.Ugent, pigs, stable hygiene

## Abstract

**Simple Summary:**

Biosecurity measures play a pivotal role in minimizing the risk of introducing and spreading infectious agents. Within external and especially internal biosecurity, cleaning and disinfection (C&D) procedures play an important role. The current study aimed to assess the implementation of C&D procedures on pig farms in Europe during 2019–2022, with a focus on identifying areas that warrant improvement. Increased C&D implementation is observed in countries like Belgium, Finland, Italy, and Spain, signaling a high level of farmers’ awareness. The study identifies stronger implementation of C&D measures in the framework of external biosecurity but notes gaps in the application of C&D measures for material introduction practices (22% of farms). In the frame of internal biosecurity, more gaps were highlighted in the presence and use of hand hygiene stations (19% of farms), boots disinfection equipment (40% of farms), cleaning between compartments, and evaluation of the efficacy of C&D measures. Notably, only 1% of farms evaluate hygiene after C&D procedures, revealing an important area for improvement. In conclusion, the analysis highlights C&D implementation in European pig farming, emphasizing progress areas and advocating for targeted awareness campaigns and training initiatives to improve biosecurity practices.

**Abstract:**

This study delves into the assessment of cleaning and disinfection (C&D) measures within the context of European pig farming, employing the Biocheck.UGent™ tool as an effective instrument for evaluation. A comprehensive set of relevant parameters was examined to enable meaningful comparisons across farms from 10 European countries during four years (2019–2022). Findings indicate a notable increase in C&D measure implementation in select countries (Belgium, Finland, Italy, and Spain), reflecting heightened awareness and responsibility among farmers. Additionally, the overall score for the C&D subcategory highlights variation across countries, with Italy (75), Poland (74), and Belgium (72) displaying the highest scores, while Ireland (56), Slovenia (55), and Serbia (50) reported the lowest scores. However, the considerable variation in the number of participating farms necessitates cautious comparisons. The study identifies well-implemented C&D measures in the frame of external biosecurity but underscores gaps in the application of C&D measures for the material introduction practices across farms (22% of farms), which are attributed to awareness gaps and resource limitations. In the areas of internal biosecurity, strong points include C&D procedures after each production cycle (79%), implementing different stages in the C&D process (65%) and sufficient sanitary break (82%), while gaps are evident in the presence and using of hand hygiene stations (19% of farms) and boots disinfection equipment (40% of farms) between compartments/units. Notably, the study reveals a lack of evaluation of hygiene after C&D procedures (only 1% of farms), signaling critical knowledge gaps among farmers regarding proper assessment tools and methods. In conclusion, this comprehensive analysis sheds light on the implementation status of C&D measures in European pig farming, offering insights into both areas of progress and those requiring improvement. The findings emphasize the need for targeted awareness campaigns and training initiatives to bolster biosecurity practices within the industry.

## 1. Introduction

Both in the framework of reducing antimicrobial usage (AMU) and preventing the spread of epidemic and endemic diseases, prioritizing preventive veterinary management strategies is crucial for the sustainability of animal health [1,2,3]. A primary approach to reducing infections and the need for treatment is establishing and maintaining biosecurity on animal farms [4,5,6,7]. Past studies indicate that improving biosecurity on livestock farms is particularly crucial in combating antimicrobial resistance as it can help to reduce the AMU as well as curb the dissemination and persistence of resistant microbes within farms [8,9,10,11].

Farm biosecurity comprises a set of measures aimed at minimizing the risk of introducing pathogenic agents (external biosecurity) and preventing the spread of these pathogenic agents within a herd (internal biosecurity) [2,3,12]. Among the various parameters of external and internal biosecurity, the role and impact of cleaning and disinfection (C&D) measures have been highlighted in various studies [13,14,15,16,17]. The C&D measures involve a series of step-by-step procedures such as dry and wet cleaning, rinsing, disinfecting, and evaluating the effectiveness of the process, along with additional measures that must be applied for premises, vehicles, equipment, personnel, visitors, protective clothing, manure, etc. [17,18,19]. Therefore, proper adherence to C&D procedures is crucial in reducing the transmission of infectious agents within and between animal farms [14,17,20,21].

In Europe, biosecurity measures on pig farms vary across countries and regions, as there are no standardized procedures or regulations [2,14,22]. Each country typically has its own national guidelines or codes of practice for biosecurity, and the implementation of these measures depends on factors like geographical location, farm size, production type, techno-managerial capabilities, and the epidemiological situation [16,17,23,24]. Moreover, there is a scarcity of data regarding the level of implementation of biosecurity measures on conventional indoor pig farms in various European countries [6,10,23,25].

To address these gaps, a specialized free tool with public access has been developed, enabling quantitative assessment of both external and internal biosecurity measures at the farm. This tool, Biocheck.UGent™, is widely used in Europe. Many field-based epidemiological studies have been conducted using this system to evaluate the biosecurity measures across farms with different animal species [4,8,26,27,28]. However, the available published data concerning C&D on pig farms in European countries remain limited. With this background, the study was conducted to assess the implementation of C&D measures and identify the current gaps in the C&D procedures on pig farms in European countries using Biocheck.UGent™ data.

## 2. Materials and Methods

### 2.1. Scoring System

Biocheck.UGent™ is a risk-based biosecurity scoring system developed by the Faculty of Veterinary Medicine at Ghent University, Belgium. This standardized tool is based on scientific research, which allows the quantitative assessment of biosecurity measures implementation on farms and thereby facilitates comparative analysis between different farms or regions or countries [26,27].

In brief, the Biocheck.UGent™ scoring system for pig’s farms consists of 109 questions, divided into six subcategories for external biosecurity and six subcategories for internal biosecurity. External biosecurity encompasses subcategories such as the purchase of breeding pigs, piglets, and semen; the transportation of animals, removal of carcasses and manure; feed, water and equipment supply; visitors and farmworkers; vermin and bird control; and location of the farm. Internal biosecurity includes subcategories such as disease management; farrowing and suckling period; nursery unit; finishing unit; measures between compartments, working lines and use of equipment; as well as cleaning and disinfection. The comprehensive information on each (sub)category can be assessed at https://biocheckgent.com/en, accessed on 31 December 2022. A score is given to each question, with 0 indicating the absence of a biosecurity measure and a score between 0.5 and 1.0, indicating the presence of measures and ranking based on their importance in disease prevention. The assigned score reflects the significance of the question [29], allowing for a comprehensive evaluation of biosecurity measures through weighted scores for each subcategory. Scores for each question are calculated using a weighting factor based on the importance of the biosecurity measure. The survey for pig indoor farms can be accessed at https://biocheckgent.com/en/questionnaires/pigs, accessed on 31 December 2022, and is available in multiple languages, including English, Dutch, French, Polish, German, Italian, Norwegian, Spanish, Vietnamese, Hungarian, and Chinese.

### 2.2. Data Collection

**Figure 1:**
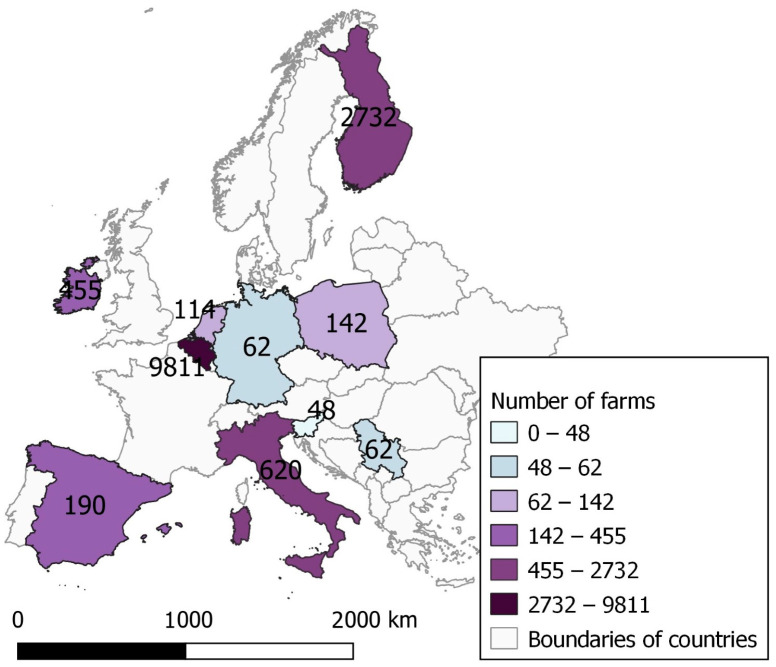
Sampling regions for 2019–2022.

Our analysis utilized the dataset sourced from the Biocheck.UGent™ database. The required permission to access the data was obtained in December 2022. The dataset encompassed responses from the voluntary online completion of the Biocheck.UGent™ questionnaire. For this study, data included between 1 January 2019 and 31 December 2022 were utilized. A precondition for incorporation into the trend analysis involves achieving a specified minimum number of observations, originating from 40 farms in total and a minimum of 20 farms on an annual basis for each country (Figure 1). In this, it has to be noted that sometimes the same farm can come back in the next year, so not all observations are necessarily different farms.

For the present study, we selected questions relevant to C&D measures for external and internal pig farm biosecurity. In total, we analyzed twelve parameters: seven specifically for the “cleaning and disinfection” subcategory (Table 1), three from “measures between compartments, working lines, and use of equipment”, one from “feed, water, and equipment supply”, and one from “visitors and farmworkers”. The analyses of the C&D measures implemented at the external biosecurity primarily revolve around assessing four key aspects: the presence and proper utilization of hygiene locks, the availability and usage of disinfection baths or boot washers, the regularity of fluid changes in the disinfection baths, and the implementation of measures for the introduction of materials. The assessment of C&D measures for maintaining internal biosecurity included measures between compartments/units, measures in corridors and loading areas, measures conducted after each production cycle, adherence to all stages of the C&D procedure, sufficient sanitary breaks, and the evaluation of the efficacy of all implemented measures.

The score for the C&D subcategory is determined by assessing various internal and external parameters (questions 3, 4, 8, 9, 10, 11, and 12) (Table 1). The risk-based biosecurity quantification system generates scores that correspond to the extent of biosecurity measures implemented, with scores ranging from 0 (indicating a complete absence of any biosecurity measures) to 100 (representing the full implementation of all available biosecurity measures).

Data analysis: The analysis of the frequency of the responses for each targeted parameter was carried out. The options “No” or “Never” were considered for non-implementation, “Yes” or “Always” for complete implementation, and “Sometimes” for partial implementation. In the Biocheck.UGent™ scoring system, the option “Sometimes” is allocated the same score as “Never”. The collected data were transformed into a Microsoft^®^ Excel 2019 (Microsoft Corp., Santa Rosa, CA, USA) for statistical analysis.

## 3. Results

By the end of December 2022, a total of 37,283 submissions from conventional indoor pig farms were recorded. A total of ten European countries viz., Belgium, Finland, Ireland, Italy, Spain, the Netherlands, Germany, Poland, Slovenia, and Serbia were included in the analysis, meeting the inclusion criteria. Among these, 14,236 observations relevant to the present study were selected, excluding ‘fictional’ data (data generated through using the Biocheck.UGent™ survey as an exercise not necessarily representing a real herd). More than half of the responses were received in 2021 (*n* = 6403). Among the surveyed countries, Belgium, Finland, Italy, and Ireland contributed the highest number of observations, indicating a major participation from these countries. On the other hand, fewer surveys were provided by Slovenia, Serbia, and Germany (Table 2). It is important to mention that in Belgium, Finland, Ireland, and Italy, the use of the Biocheck.UGent™ score system for the evaluation of conventional indoor pig farms is included in a national biosecurity evaluation procedure, requiring annual mandatory completion.

### 3.1. Analysis of Responses for C&D

**Figure 2:**
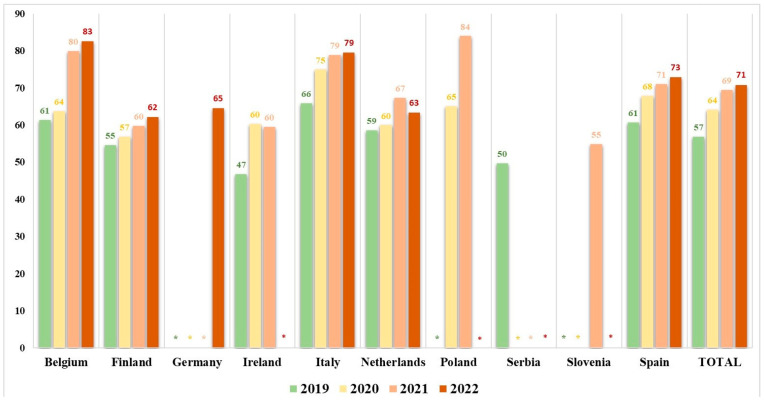
The mean score for the C&D subcategory by year per country. *—not used for evaluation of the C&D subcategory score as only years with at least 20 observations were included.

The study revealed notable variations in the mean scores for the C&D subcategory across different countries and responding years (Figure 2). Italy (75), Poland (74), and Belgium (72) exhibited the highest overall scores, while Serbia (50), Slovenia (55), and Ireland (56) reported the lowest scores during 2019–2022.

**Figure 3:**
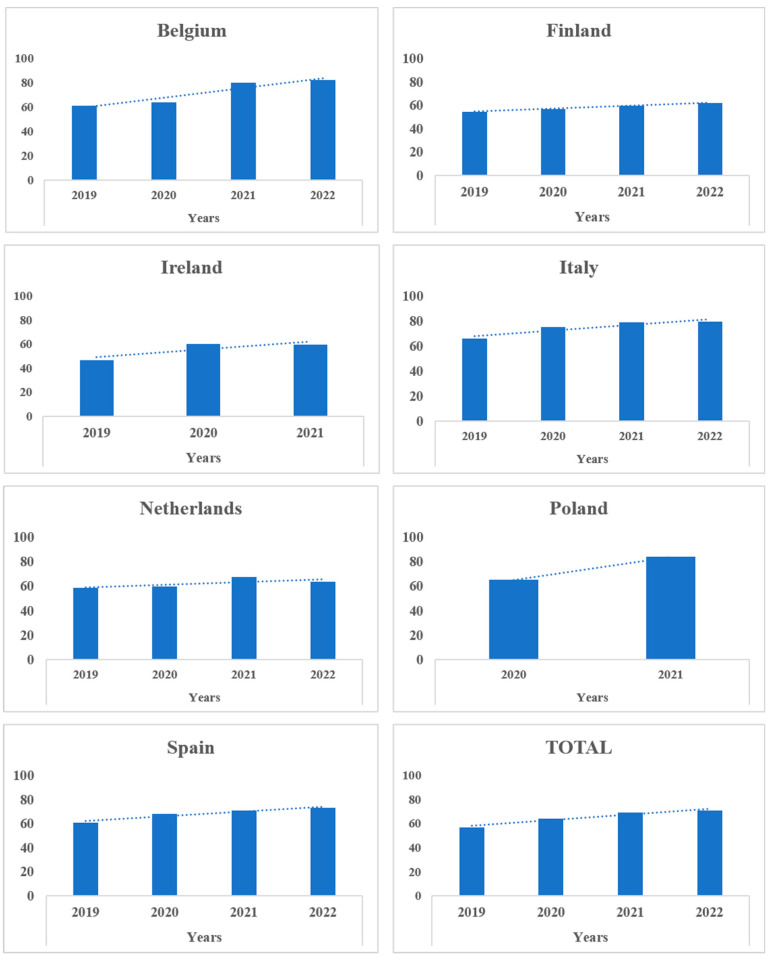
The trend of the mean score for the C&D subcategory over the four-year period per country (evaluation of the trend of the mean scores for the C&D subcategory in a 4-year period specifically focused only on countries with a minimum of 2 years of data (with at least 20 observations included)).

Over the four-year period, a general improvement in C&D measures can be seen, especially in Belgium, Finland, Italy, and Spain. The Netherlands demonstrated an increase in the level of C&D measures from 2019 to 2021 but experienced a decrease in scores from 2021 to 2022. Other countries, such as Ireland and Poland, also demonstrated an increase in C&D scores over several years (Figure 3).

### 3.2. C&D Measures for External Biosecurity

The analyses of the C&D measures implemented at the external biosecurity are provided in Table 3.

The findings regarding the ‘presence of hygiene locks and consistent usage of them’ by visitors upon entering the stables have shown a good level of application, with 74% (*n* = 11,866) of respondents confirming adherence to this measure. The data from Table 3 also suggest that the implementation of these measures has been successful in most countries, especially in the Netherlands (94%, *n* = 107) and Germany (94%, *n* = 58), contrary to Slovenia, where only 27% (*n* = 13) of respondents reported the presence of a hygiene lock and its utilization by visitors.

The results of the ‘presence and proper usage of disinfection baths or boot washers’ at the entrance of farms indicate that in 55% (*n* = 11,511) of cases, the farms had disinfection baths or boot washers installed and they were being appropriately utilized. The observations indicate that these measures were well implemented in Belgium (88%, *n* = 8614), Finland (79%, *n* = 2146), and Poland (75%, *n* = 107). Conversely, the implementation of these measures was found to be inadequate in Slovenia (19%, *n* = 9) and Italy (30%, *n* = 183).

The outcomes pertaining to the ‘regularity of fluid changes in disinfection baths’ revealed a well-implemented practice in all countries. Overall, 74% of farms (*n* = 9629) reported promptly changing the disinfectant upon visual contamination. Interestingly, Italian (98%, *n* = 178) and Irish (92%, *n* = 208) farms demonstrated the highest level of implementation in this regard.

The observations regarding the implementation of measures for the ‘introduction of materials’ revealed that compliance with the application of specific measures (e.g., cleaning and disinfection, quarantine period at a specific location) was noted in 22% (*n* = 2846) of farms. The Polish farms observed a higher level of implementation of these measures (65%, *n* = 93) as compared to other countries.

### 3.3. C&D Measures for Internal Biosecurity

The assessment of C&D measures for maintaining internal biosecurity is presented in Table 4.

It was observed that the ‘protocol for C&D of equipment after use’ was present in almost half of the farms (51%, *n* = 7420), with higher implementation levels among Serbian farms (77%, *n* = 48). However, inadequate implementation of the practices was identified regarding ‘hand and boot cleaning and disinfection between compartments/units’ in most countries. Only 40% of farms (*n* = 5124) reported the presence of disinfection baths and/or boot washers or the practice of changing boots, and even fewer farms (19%, *n* = 3090) had hand washing stations and/or hand disinfection equipment between compartments/units. Notably, Polish farms showed the best implementation for these practices (68%, *n* = 96 and 49%, *n* = 69, respectively).

The assessment also emphasized the importance of ‘implementing C&D measures in corridors and loading areas following pig movement’. Over half of farms (60%, *n* = 9974) confirmed the implementation of these measures, with Italian (87%) and Belgian (78%) farms demonstrating adequate performance.

In terms of implementing ‘C&D procedures after each production cycle’, overall, 79% of farms (*n* = 9920) reported compliance, with Spanish (97%, *n* = 184), Serbian (97%, *n* = 60), Polish (96%, *n* = 136), Italian (96%, *n* = 594), and German (92%, *n* = 57) farms showing the high rates of implementation.

More than half of farms (65%, *n* = 11,516) reported following proper C&D procedures, primarily observed on Italian, Belgian, and Polish farms. These procedures demonstrated maintaining hygiene standards, incorporating ‘different stages of C&D process’ (such as dry cleaning, soaking, high-pressure cleaning, the first drying of the stable, disinfection of the stable, the second drying of the stable, and testing of the efficiency of the procedure) and allocating sufficient time for each stage. The majority of farms (82%, *n* = 13,382) reported having enough time for drying and adjusting the temperature before pigs entered the stables. However, the effectiveness of all C&D practices was only validated by 1% of the farms (*n* = 218).

## 4. Discussion

The Biocheck.UGent™ tool surveys are effective instruments for evaluating the execution of C&D measures. The tool offers a comprehensive set of relevant parameters, enabling meaningful comparisons across farms, regions, and countries. However, we have to take into account the possible presence of biases in the data used. Firstly, the submissions could have originated from the best-performing farms, potentially leading to an overestimation of the overall biosecurity measures, especially in those countries where the Biocheck.UGent™ scoring system was used on a voluntary basis (Germany, the Netherlands, Serbia, Slovenia, Spain, and Poland). In these countries, it is also possible that the submissions might have come from specific regions within a country, which may not accurately represent the biosecurity practices of all farms nationwide. The latter risk is not so much present in those countries (Belgium, Finland, Ireland, and Italy) where Biocheck.UGent™ biosecurity evaluations have become part of a mandatory national auditing campaign. In these countries, the data provide a more or less complete overview of the actual situation. Yet, on the other hand, here we have the risk that some respondents provide answers that are better than the real situation and rather reflect what they believe they are to respond instead of what is really applied. Moreover, it is crucial to acknowledge that the responses to the survey may vary based on the respondent’s profile, such as being a veterinarian, practitioner, farmer, etc. In our specific context, in countries with mandatory national auditing campaigns, the survey for pig farms is typically completed by veterinarians. However, in countries with voluntary participation, the surveying of pig farms is generally completed by farmers in collaboration with the vets/advisors, which mitigates potential bias. In addition, socio-economic considerations may contribute to the varying levels of C&D measure implementation and correspondingly influence survey responses. Nonetheless, our dataset presents substantial advantages primarily attributed to the validated methodology, ensuring a high degree of accuracy and reliability. In addition, our analysis is based on data from a huge database, affording us the capacity to check and determine the level of the implementation of C&D measures within European countries at the vanguard of pig industry production and research [4,27,30]. The overall findings highlight both areas of progress and areas that require improvement, offering opportunities to strengthen external and internal biosecurity measures and reduce the risk of disease transmission within the pig farming industry.

In general, the analysis of the C&D subcategory scores revealed an upward level of implementation measures in the majority of the included countries. This may be the result of an increasing level of awareness and responsibility on farms. It may also demonstrate a biosecurity audit, wherein farmers and veterinarians try to improve the situation by working on the biosecurity gaps [8,10,31].

The overall score for the C&D subcategory ranged from 47 to 84, with the highest scores observed in Italy, Poland, and Belgium. Our results are in agreement with the results provided by Chantziaras et al. (2020) on the biosecurity levels of pig fattening farms from four EU countries (Belgium, the United Kingdom, Finland, and Poland), where the score for the C&D subcategory ranged from 40 to 87, and the highest scores for C&D measures were observed in Belgian and Polish farms [32].

Our study also revealed that there were more deficiencies in implementing C&D measures in the framework of internal biosecurity as compared to external biosecurity. This observation may be explained by the fact that farmers find it easier to enforce rules on others (e.g., visitors, vehicles), which primarily relate to external biosecurity, as opposed to changing their own habits, which are more closely linked to internal biosecurity [4,20].

The study findings regarding the C&D measures for maintaining external biosecurity indicated well-implemented measures with regard to the presence of hygiene locks and their consistent usage by visitors, the presence of footbaths, and regular fluid changes in disinfection baths in most countries, except for Serbia and Slovenia. The measures for the introduction of materials (such as cleaning and disinfection) were poorly applied across farms in all countries except Poland (65%).

The assessment of C&D measures related to internal biosecurity revealed strong points as well as some gaps. Strong points were observed with regard to the implementation of the different stages in the C&D process, conducting C&D after each production cycle, C&D measures in corridors and loading areas, and long enough sanitary breaks. However, important gaps were observed regarding the presence of hand washing stations and/or hand disinfection equipment between compartments/units (19% in total), particularly in Serbia (5%), Germany (10%), Spain (11%), Finland (12%), and the Netherlands (12%); only Polish farms (49%) exhibited better implementation. In addition, fewer but also noteworthy gaps were identified regarding the presence of disinfection baths or boot washers between compartments or units, with a total of only 40% of the farms applying these measures. This tendency suggests a considerable need for improvement in maintaining proper biosecurity measures between compartments.

Finally, an important shortcoming was observed in the assessment of the effectiveness of C&D measures, with only 1% of farms reporting that they conducted evaluations of hygiene after C&D procedures (e.g., with hygienogram). This may be linked to the limited information available to farmers regarding proper tools or methods for evaluating hygiene status in animal houses. As reported by Alarcón et al. (2021), pig farmers often adopt the assessment of the effectiveness of hygiene procedures with low perseverance and consistency [20]. Practical challenges, including resource availability, time constraints, and complex data collection methods, may hinder comprehensive assessments [33]. Yet, the effect of the limited evaluation of the quality of the C&D procedures is that farmers and farm workers often have no clue of whether they are performing the procedures sufficiently well. Designing farm-specific assessment approaches can lead to more accurate evaluations by considering factors like farm size, layout, disease risks, and biosecurity measures [20,21,34,35].

In general, motivating farmers to adopt a standard hygiene protocol and alter their daily routines poses a well-known challenge [23,36,37]. As reported by Garforth et al. (2013) many farmers believe they are already taking all reasonable measures to minimize disease risk, considering other practices irrelevant [38]. Additionally, limited resources, such as insufficient supplies of cleaning agents, disinfectants, personal protective equipment, or appropriate infrastructure for quarantine areas, can make it challenging to adhere to the recommended protocols [23,36,39]. As reported by Alarcón et al. (2021) farm personnel play a critical role in maintaining internal biosecurity, with responsibilities for implementing biosecurity rules while also having the potential to inadvertently spread pathogens within the farm [20]. Cross-contamination risks may not be fully understood by farmers and employees, leading to inadequate hand and boot cleaning before moving to different parts of the farm, especially in small herds [2,40,41,42]. In addition, time restrictions may cause them to speed up or completely omit phases in the hygiene procedure [43]. Thereby, inconsistent practices, lack of time, and inadequate training contribute to these issues, increasing disease transmission risk [2,20,23]. Recent studies have found that non-compliance with biosecurity measures is often related to inadequate training of farm personnel and poor communication with advisors [36,44,45,46]. Additionally, factors like farm infrastructure, condition variability, insufficient supervision, monitoring, and documentation also contribute to the gaps [34,47,48]. A study conducted by Chen et al. (2023) highlighted that biosecurity cognition plays a substantial mediating role in the adoption of C&D methods by pig farmers who have undergone specialized training [49]. Therefore, addressing all these factors through improved knowledge dissemination and effective evaluation techniques can enhance biosecurity practices on pig farms [17,36,45].

## 5. Conclusions

The assessment of C&D measures on conventional indoor pig farms reveals both areas of progress and scope for improvement. While some farms demonstrate commendable commitment to hygiene standards, there is a need for enhanced practices in areas such as hand and boot cleaning between compartments/units, verification of effectiveness, and the implementation of C&D measures across all compartments and units. Sharing best practices and promoting knowledge exchange among countries can contribute to elevating biosecurity standards industry-wide. By addressing these key findings and implementing targeted interventions, farmers can strengthen biosecurity measures, safeguard the animal health, and mitigate the risk of disease transmission.

## Figures and Tables

**Table 1:** Assessment of parameters for C&D measures * on pig farms.

External Biosecurity Measures	Internal Biosecurity Measures
Is there a hygiene lock available and is it always used by visitors when they enter the stables? Are specific measures taken for the introduction of material (e.g., cleaning and disinfection, quarantine period at a specific location)? Are disinfection baths/boot washers present at the entrance of the farm and are they used? Is the fluid of the disinfection baths immediately changed when visually contaminated?	5. Are hands washed and/or disinfected between different compartments/units? 6. Are either disinfection baths and/or boot washers used between different compartments/units or are boots changed between compartments? 7. Is there a protocol for the cleaning and disinfection of equipment (such as brooms, spades) after their use and is this protocol abided by? 8. Are the stables/compartments cleaned and disinfected after each production cycle? 9. Are the corridors and the loading area cleaned and disinfected after pigs are moved? 10. Is the sanitary break long enough, i.e., is there sufficient time for drying and adjusting the temperature before pigs enter the stable? 11. Are the different stages in the cleaning and disinfection process respected and is there sufficient time (according to the used product specifications) provided for each stage? 12. Is the efficacy of cleaning and disinfection checked with for example a hygienogram?

* questions 3, 4, 8, 9, 10, 11 and 12 are included in the cleaning and disinfection subcategory. The answers to these questions are used to calculate the C&D score.

**Table 2:** Number of responses per country from 2019 to 2022.

No.	Countries	2019	2020	2021	2022	Total
1	Belgium	93	45	4971	4702	9811
2	Finland	398	674	871	789	2732
3	Italy	85	64	120	351	620
4	Ireland	116	151	182	6 *	455
5	Spain	34	45	87	24	190
6	Poland	1 *	21	108	12 *	142
7	The Netherlands	22	34	27	31	114
8	Germany	17 *	9 *	9 *	27	62
9	Serbia	51	0 *	6 *	5 *	62
10	Slovenia	0 *	0 *	41	7 *	48
	Total	817	1043	6422	5954	14,236

*—not used for evaluation of the C&D subcategory score, as only years with at least 20 observations were included.

**Table 3:** Percentage of farms with C&D measures in the frame of external biosecurity (2019–2022) (The color gradient in the illustration represents the percentage of farms implementing C&D measures. The intensity of green indicates a high level of implementation, yellow signifies a medium level, and red indicates a low level).

	Country	1. Presence of Hygiene Lock	2. Presence of Disinfection Baths/Boot Washers Present at the Entrance of the Farm	3. Appropriate Change of Fluid in Disinfection Baths *	4. C&D Measures Taken for the Introduction of Materials
1	Belgium	85%	88%	89%	21%
2	Finland	88%	79%	60%	19%
3	Germany	94%	42%	69%	19%
4	Ireland	56%	49%	92%	13%
5	Italy	77%	30%	98%	11%
6	The Netherlands	94%	61%	67%	19%
7	Poland	87%	75%	82%	65%
8	Serbia	55%	55%	26%	16%
9	Slovenia	27%	19%	78%	21%
10	Spain	79%	52%	79%	19%
	Average	74%	55%	74%	22%

*—only applicable to farms that reported the presence of disinfection baths.

**Table 4:** Percentage of farms with C&D measures in the frame of internal biosecurity (2019–2022) (The color gradient in the illustration represents the percentage of farms implementing C&D measures. The intensity of green indicates a high level of implementation, yellow signifies a medium level, and red indicates a low level).

	Country	1. Presence of Protocol for the C&D of Equipment	2. Presence of Hand Washing Stations and/or Hand Disinfection Equipment between Compartments/Units	3. Presence of Disinfection Baths/Boot Washers between Compartments/Units	4. C&D Measures in Corridors and Loading Areas	5. Conducted C&D after Each Production Cycle	6. Provided Different Stages in the C&D Process	7. Long Enough Sanitary Break	8. Checking the Efficacy of C&D
1	Belgium	57%	24%	38%	78%	74%	89%	96%	2%
2	Finland	34%	12%	27%	43%	49%	58%	90%	0%
3	Germany	58%	10%	32%	52%	92%	53%	74%	0%
4	Ireland	46%	20%	35%	51%	45%	61%	87%	1%
5	Italy	56%	31%	38%	87%	96%	95%	96%	2%
6	The Netherlands	47%	12%	54%	61%	82%	61%	69%	1%
7	Poland	49%	49%	68%	64%	96%	87%	92%	2%
8	Serbia	77%	5%	42%	69%	97%	23%	63%	2%
9	Slovenia	25%	21%	17%	50%	65%	56%	63%	0%
10	Spain	56%	11%	46%	47%	97%	67%	89%	2%
	Average	51%	19%	40%	60%	79%	65%	82%	1%

## Data Availability

The data presented in this study are available upon request from the corresponding author. The data are not publicly available due to privacy and confidentiality agreements with the participants.
